# Type V Collagen Induced Tolerance Suppresses Collagen Deposition, TGF-β and Associated Transcripts in Pulmonary Fibrosis

**DOI:** 10.1371/journal.pone.0076451

**Published:** 2013-10-21

**Authors:** Ragini Vittal, Elizabeth A. Mickler, Amanda J. Fisher, Chen Zhang, Katia Rothhaar, Hongmei Gu, Krista M. Brown, Amir Emtiazdjoo, Jeremy M. Lott, Sarah B. Frye, Gerald N. Smith, George E. Sandusky, Oscar W. Cummings, David S. Wilkes

**Affiliations:** 1 Center for Immunobiology, Indiana University School of Medicine, Indianapolis, Indiana, United States of America; 2 ImmuneWorks Inc., Indianapolis, Indiana, United States of America; 3 Department of Pathology, Indiana University School of Medicine, Indianapolis, Indiana, United States of America; Helmholtz Zentrum München/Ludwig-Maximilians-University Munich, Germany

## Abstract

**Rationale:**

Idiopathic pulmonary fibrosis (IPF) is a fatal interstitial lung disease characterized by progressive scarring and matrix deposition. Recent reports highlight an autoimmune component in IPF pathogenesis. We have reported anti-col(V) immunity in IPF patients. The objective of our study was to determine the specificity of col(V) expression profile and anti-col(V) immunity relative to col(I) in clinical IPF and the efficacy of nebulized col(V) in pre-clinical IPF models.

**Methods:**

Col(V) and col(I) expression profile was analyzed in normal human and IPF tissues. C57-BL6 mice were intratracheally instilled with bleomycin (0.025 U) followed by col(V) nebulization at pre-/post-fibrotic stage and analyzed for systemic and local responses.

**Results:**

Compared to normal lungs, IPF lungs had higher protein and transcript expression of the alpha 1 chain of col(V) and col(I). Systemic anti-col(V) antibody concentrations, but not of anti-col(I), were higher in IPF patients. Nebulized col(V), but not col(I), prevented bleomycin-induced fibrosis, collagen deposition, and myofibroblast differentiation. Col(V) treatment suppressed systemic levels of anti-col(V) antibodies, IL-6 and TNF-α; and local *Il-17a* transcripts. Compared to controls, nebulized col(V)-induced tolerance abrogated antigen-specific proliferation in mediastinal lymphocytes and production of IL-17A, IL-6, TNF-α and IFN-γ. In a clinically relevant established fibrosis model, nebulized col(V) decreased collagen deposition. mRNA array revealed downregulation of genes specific to fibrosis (*Tgf-β, Il-1β, Pdgfb*), matrix (*Acta2, Col1a2, Col3a1, Lox*, *Itgb1/6, Itga2/3*) and members of the TGF-β superfamily (*Tgfbr1/2, Smad2/3, Ltbp1, Serpine1, Nfkb/Sp1/Cebpb*).

**Conclusions:**

Anti-col(V) immunity is pathogenic in IPF, and col(V)-induced tolerance abrogates bleomycin-induced fibrogenesis and down regulates TGF- β-related signaling pathways.

## Introduction

Idiopathic Pulmonary Fibrosis (IPF) is a fatal disease with no effective treatment and a three year mean survival rate [1]. The early IPF lesion is believed to be alveolitis that leads to thickening of the alveolar wall and epithelial cell death, eventually culminates in fibrogenic foci which is characterized by overexpression of collagen types I [col(I)] and V [col(V)] [2]. However, the relative quantities of total col(I) to col(V) and their respective individual chains in the IPF lung are unknown.

Type V collagen ([col(V)] is a minor collagen normally sequestered within the lung interstitium and therefore, hidden from the immune system. Remodeling, a characteristic feature of IPF, leads to col(V) exposure and overexpression which in turn is associated with the development of anti-col(V) immunity - indicating an autoimmune aspect in IPF pathogenesis. Specifically, up to 60% of IPF patients have anti-col(V) reactive T cells [3], and nearly half develop specific systemic antibody responses [4].

Persistent immune responses and resulting dysregulated tissue healing accelerates the pathogenic conditions towards fibrosis and accumulation of extracellular matrix components [5]. When autoimmune or other agents cause epithelial injury, matrix metalloproteinases [6] are expressed to gain access to damaged tissues. Subsequently, pro-inflammatory chemokines and growth factors are recruited to the site of injury. During this phase, the chemokine cocktail leads to myofibroblast accumulation and deposition of extracellular matrix components such as integrins [7]–[9], fibronectins [10] and collagens [11]. TGF-β, a master regulator of the fibrogenic process [12], is intricately involved in the crosstalk of other pro-fibrotic molecules, particularly PDGF [13] and IL-1β [14]. In this pro-fibrotic environment, intricate interactions which exist between specific key players, for example - TGF-β and integrins, accelerate fibrogenesis. Integrins, which are receptors for cell-cell and cell-matrix adhesion, has been reported to activate latent TGF-β [8], specifically αVβ6 integrins. Similarly, TGF-β has been reported to upregulate integrin expression [15]. Notably, collagens may bind to α3β1 integrins via the RGD binding site, since it is recognized in all ligands [16]. Collagen type I has been reported to bind to α3β1 integrins via the DGEA binding site [17]. Interestingly, the effects of col(V)-induced tolerance on the gene expression of integrins and other fibrosis related genes, in IPF are unknown.

Col(V)-induced tolerance reportedly downregulates TGF-β in an experimental model of systemic sclerosis [18]. Col(V)-induced tolerance by systemic administration abrogates obliterative bronchiolitis (OB) post lung transplant [19] and bleomycin-induced acute injury [20]. Since col(V) is overexpressed in IPF and associated with anti-col(V) immunity, then in the current study we tested the hypothesis that nebulizing col(V) to significantly scarred lungs will arrest progression of fibrosis and that col(V)-induced tolerance by inhalation will attenuate pro-fibrotic signaling in a pre-clinical model of IPF. Our findings suggest that col(V)-induced tolerance effectively mitigates lung fibrosis and down regulates TGF-β and related signaling pathways.

## Materials and Methods

### Human Studies

All protocols were approved by the Institutional Review Board, IUSM.

Plasma and frozen tissues from IPF patients were obtained from the Lung Tissue Research Consortium (LTRC) sponsored by NIH/NHLBI. The LTRC collected medical data and tissues from large cohorts of patients to study the cause, early detection and best treatment for IPF and other similar lung diseases. A written consent was obtained from patients by LTRC and this questionnaire is available at their public domain http://www.ltrcpublic.com/docs/LTRC_Consent_Jul_2010.pdf. Plasma from IPF patients were also provided by ImmuneWorks, Inc. The samples were collected by written consent from patients enrolled in a clinical trial. The patient selection criteria are available in the public domain: http://www.clinicaltrials.gov/ct2/show/NCT01199887?term=pulmonaryfibrosisimmuneworks&rank=1. Therefore, the individuals in this manuscript have given written informed consent (as outlined in PLOS consent form) to publish these research details. Pathologist-certified paraffin-embedded lung tissues and normal lung specimens were also procured from the Department of Pathology, Indiana University School of Medicine (IUSM).

### Real Time PCR

Total RNA was isolated from two 20 µm scrolls of formalin-fixed paraffin embedded tissue using RecoverAll™ Total Nucleic Acid Isolation Kit (Ambion) according to manufacturer's instructions. cDNA was synthesized using the iScript cDNA synthesis kit (Bio-Rad, Hercules, CA). Alpha 1 and 2 chains for col(I) and col(V) and GAPDH were performed using the Assays-on-Demand™ gene expression kits (Applied Biosystems, Foster City, CA) on a Smartcycler (Cepheid, Sunnyvale, CA). qRT-PCR for *Il-17a* and *β-actin* in mouse whole lung homogenates were performed using the following real-time primer sequences: β-actin- FW:CAATAGTGATGACCTGGCCGT, RV:AGAGGGAAATCGTGCGTGAC, IL-17A- FW:CTGTGTCTCTGATGCTGTTG, RV:ATGTGGTGGTCCAGCTTTC.

### Mouse Fibrosis PCR Microarrays

Murine lung mRNA was used to generate cDNA. The Mouse Fibrosis PCR array- RT^2^ Profiler PCR Arrays version 3.0 (SABiosciences, Qiagen, Valencia, CA) was used as per manufacturer's instructions and array data were analyzed using Qiagen PCR Array Data Analysis software. The array data is provided in Table S1.

### Circulating col(V) and col(I) antibody detection

Known volumes of plasma (10 µl) were incubated with microsphere beads coated with col(V) or col(I) and the signal was detected by flow cytometry as previously described [21]. Results of the duplicate reactions were expressed as Mean Fluorescent Intensity (MFI) as a fold change compared to pooled normal plasma.

### Pepsin digestion

Frozen IPF and normal lung tissues were homogenized and subjected to pepsin extraction by lysing the tissues in a buffer containing pepsin in 0.2M acetic acid. The lysates were then subjected to dialysis, lyophilization, followed by careful weighing and re-suspension in lysis buffer at a concentration of 1 mg/ml. Pepsin-digested samples were equally loaded at 15 µg in a fixed 5% concentration agarose gel and electrophoresed as previously described [22]. Densitometry measurements: Bands corresponding to the individual chains of col(V) or col(I) was measured in normal and IPF lungs using NIH Image J. The col(V) protein standard was used as a control on the exposure time. Values were then expressed as Mean ± SEM.

### Animal Studies

The Indiana University School of Medicine Animal Care and Use Committee (IACUC) approved the animal protocols used in this study. Female C57BL6 mice (6–8 weeks of age) were purchased from Jackson Laboratory (Bar Harbor, ME). After a 5 day period of acclimatization, mice were administered intratracheal PBS or bleomycin (0.025 U) as previously described [23]. Based on previous studies, we used varying doses of col(V): 4.16, 5.5 and 8.33 mg/kg bodyweight, at a final volume of 100 µl, and nebulized it to a mouse of average body weight of 18 gm. Mice were nebulized in a conscious state using Aeroneb® Lab Nebulizer, which is designed to produce highly respirable aerosol particles with sizes averaging between 2.5 µm–4.0 µm. Historically, aerosolized drug particles with a size of ∼3.0 µm has been documented as optimal for penetration, retention and maximal therapeutic effectiveness in the lung [24]. Lungs were processed for immunohistochemical (IHC) staining or stored at −20°C until further analyses.

### Statistical analysis

Statistical analysis was performed using Student's *t* test, one-way ANOVA with Bonferroni *post hoc* test using GraphPad Prism version 3.0 for Windows GraphPad Software (San Diego, CA, www.graphpad.com). Statistical significance was defined at *p*<0.05.

Additional descriptive material for methods used in this study is provided in File S1. The raw array data for the array analyses is provided in Table S1.

## Results

### Col(V) protein and mRNA overexpression in the fibrotic foci of lungs with IPF

**Figure 1:**
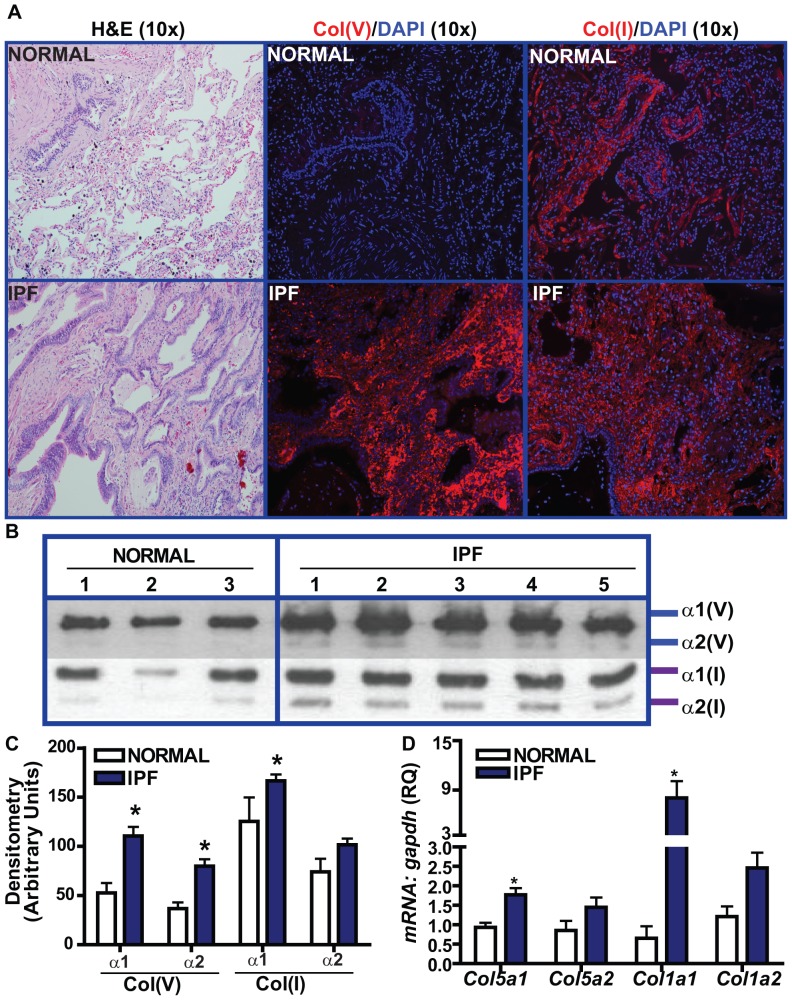
Relative expression of col(I) and col(V) in patients with UIP/IPF and pathologically normal specimens. (**A**) Lung tissue sections from UIP/IPF patients and pathologically normal specimens were immunostained with col(I) and col(V) antibodies and their IgG, followed by incubation with rhodamine-anti-rabbit. Nuclei were counterstained with DAPI. (Original magnification, 10×, representative of 4 patients). Corresponding H&E staining is also shown. (**B**) Pepsin digested lung homogenates (15 µg) and corresponding standards run in a 5% gel and immunoblotted with antibodies against col(V) and col(I). Image shows representative 3 normal and 5 IPF tissues, (**C**) Densitometry of protein expressions of individual alpha chains of col(I) and col(V) obtained from IPF lung biopsies and pathologically normal specimens. Values represent mean ± SEM of5 normals and 20 IPF specimens (p<0.01; one-way ANOVA, post hoc test: Bonferroni), (**D**) mRNA expression were determined by qPCR of lung tissue sections of UIP/IPF and pathologically normal specimens. Values represent mean ± SEM; 3 normals and 4 UIP/IPF specimens; (p<0.01; one-way ANOVA, post hoc test: Bonferroni).

Col(V) exists in the lung as the predominant heterotrimer, [α1(V)]_2_. α2(V). The activity of this molecular form to bind to heparin at physiological salt concentrations may be attributed to a proteolytic NH_2_-terminal 30-kDa fragment of the α1(V) chain [25]. Parra and colleagues have demonstrated the distribution of col(V) in IPF [26]. However, the relative quantitative distribution of col(I) to col(V) and the expression pattern of the individual chains of col(V) and col(I) in the IPF lungs are largely uncertain. In contrast to normal lungs wherein we observed strong expression of total col(I), but not col(V), in the interstitium; both col(I) and col(V) were expressed heavily in IPF lungs (Figure 1A). We observed a similar trend when we analyzed pepsin-digested normal and IPF lungs for protein expression levels of alpha 1 [α1(V)] and alpha 2 [α2(V)] chains of col(V) (Figure 1B). The expression levels of α1(V) and α2(V) were ∼2.5–3 fold higher than the normal tissues. Notably, α1(V) levels were higher than α2(V) in the IPF lungs (*p*<0.01). Similarly, α1(I) levels were higher in IPF patients compared to normal donors. IPF lungs had higher α1(I) levels than α1(V) (*p*<0.0001) (Figure 1 B,C; Figure S1). At the transcript level, we observed higher expression of α1(V) (*Col5a1*) and α1(I) (*Col1a1*) in the IPF lungs compared to normal and that *Col1a1* was ∼6 fold higher than *Col5a1* in IPF (Figure 1D). Collectively, these studies demonstrate that col(V), particularly α1(V), is overexpressed at the transcript and protein level in IPF.

### Circulating col(V)-specific antibody responses in IPF

**Figure 2:**
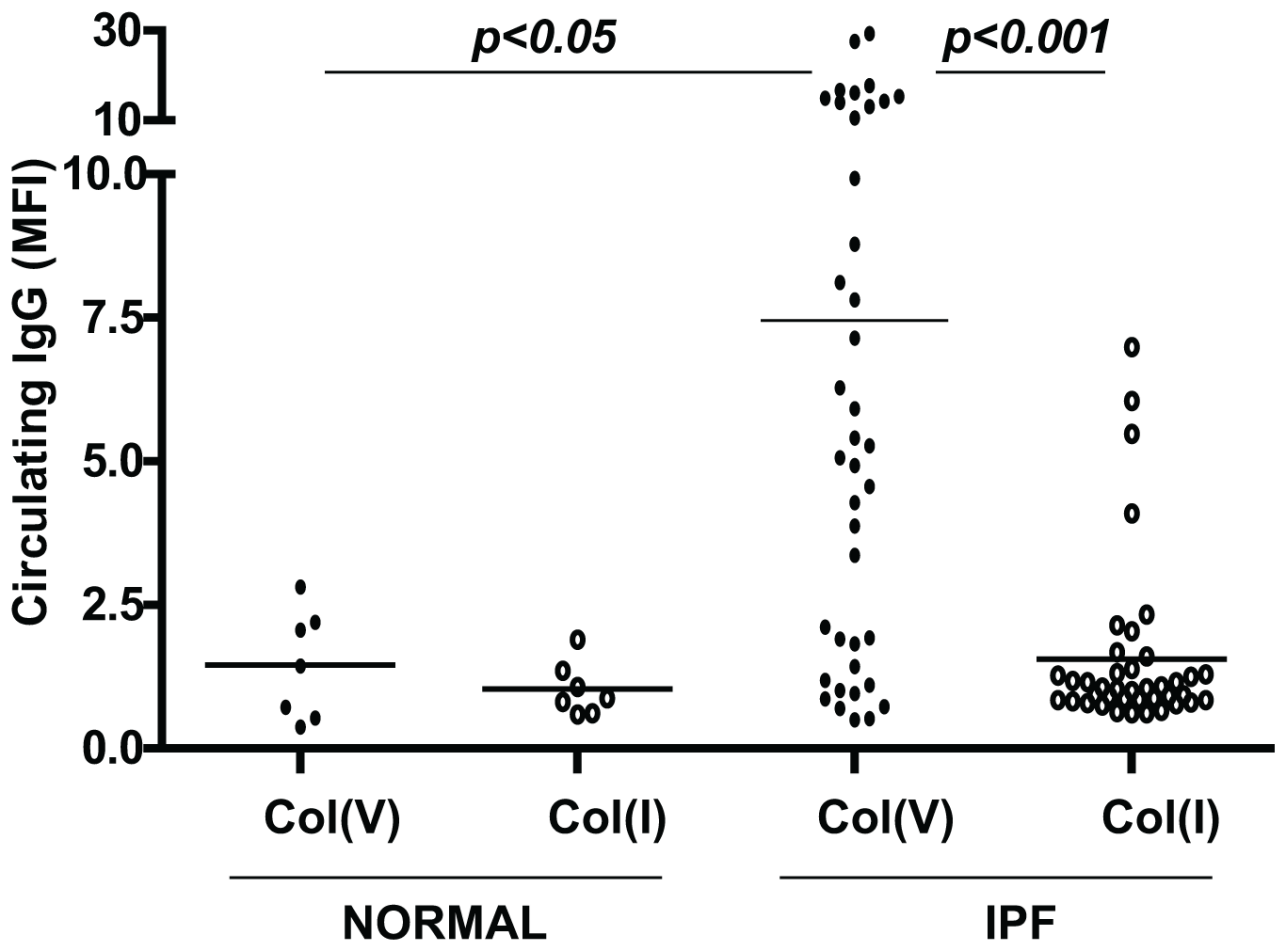
Auto-antibody responses of col(I) and col(V) in plasma of patients with UIP/IPF and normal volunteers. Col(I) and col(V) antibodies were detected in the plasma by flow cytometry in plasma of patients with UIP/IPF with varying degrees of disease severity (n = 40) and healthy normal volunteers (n = 7). Values are represented as mean ± SEM; *, p<0.05, **, p<0.001; one-way ANOVA, post hoc test: Bonferroni.

Although we have reported anti-col(V) cellular immunity in IPF [3], the relative circulating antibody levels of col(V) and col(I) are unknown. We investigated a cohort of 40 patients diagnosed with IPF as per ATS criteria as previously described [3]. The cohort has a gender distribution of 15 females and 25 males and their average age is 64.3 years with a standard deviation of 7.78. We observed that compared to normal healthy volunteers, anti-col(V) levels were higher in IPF patients (Figure 2) (*p*<0.05). Additionally, anti-col(I) antibodies were detected at very low levels in all patients studied. The above studies demonstrate that IPF is associated with systemic anti-col(V) antibodies specifically.

### Bleomycin-induced fibrosis results in col(V) overexpression and production of anti-col(V) antibodies

**Figure 3:**
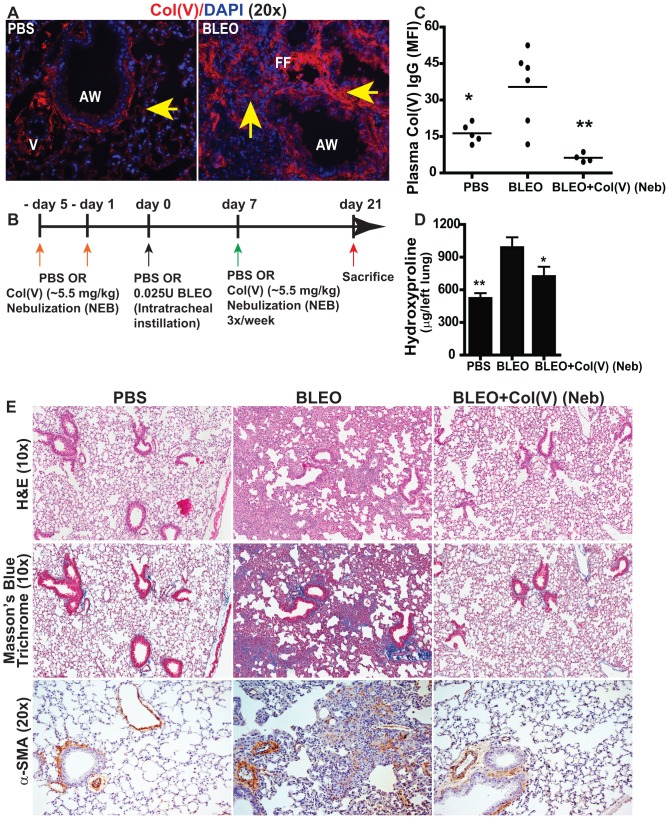
Tolerance induction of Col(V) protects against bleomycin-induced fibrosis. (**A**) Col(V) overexpression was detected in 21 day post bleomycin injury by labeling with rhodamine and counterstained with DAPI. (Original magnification, 10×). (**B**) Schematic illustration of the experimental design. (**C**) Circulating antibodies specific to col(V) were detected in 21 day post bleomycin injury mice. Values represent mean ± SEM; number of animals: PBS = 5, BLEO = 6 and BLEO+col(V) Neb = 4. Compared to bleomycin group, * p<0.001; ** p<0.01; one-way ANOVA, post hoc test: Bonferroni. (**D**) Collagen deposition was measured quantitatively by assaying for hydroxyproline concentrations from the whole left lung day 21 post bleomycin injury+col(V) treatment. Values represent mean ± SEM; n = 7 mice/group. Compared to bleomycin group, ** p<0.01; * p<0.05 one-way ANOVA, post hoc test: Newman-Keuhl's. (**E**) At day 21 post bleomycin injury, lung tissue sections were stained for H&E and Masson's blue trichrome staining. Extensive collagen deposition was observed with bleomycin injury while col(V) nebulized lungs, similar to normal lungs, had collagen deposition around airways and vasculature. Original magnifications: 10×. (Figure S2A: 1×). Lung tissue sections were immunostained against alpha-smooth muscle actin (α-SMA) or IgG. Streptavidin-conjugated horseradish peroxidase was used with 3,3′-diaminobenzidene as substrate (brown) and nuclei were counterstained with hematoxylin (blue). Original magnifications: 20×.

We next determined if the bleomycin-induced lung injury, as a model for IPF, results in col(V) overexpression, and anti-col(V) cellular and humoral immunity. Indeed, bleomycin-induced fibrosis was associated with overexpression of col(V) (Figure 3A), and development of anti-col(V) antibodies (Figure 3C) in a manner similar to that observed in clinical IPF. Since we have observed data showing that col(V) is the target of an autoimmune response in IPF in both pre-clinical and clinical studies, we next determined if col(V) could be utilized as a tolerogen to prevent fibrosis. Braun and colleagues have demonstrated that systemic administration of col(V) inhibits bleomycin-induced acute lung injury using measures of lung inflammation [20]. Since introducing antigens to the lung mucosa may result in tolerance, we nebulized col(V) into the lungs of mice using the experimental model of chronic bleomycin injury as illustrated in Figure 3B, followed by a systematic assessment of parameters associated with fibrosis. Compared to bleomycin-injured mice, nebulizing col(V) resulted in a reduction in anti-col(V) antibodies (Figure 3C), hydroxyproline concentration (Figure 3D), reduced connective tissue deposition as shown by trichrome staining and blunted myofibroblasts differentiation as observed by expression for α-smooth muscle actin (α-SMA) (Figure 3E).

**Figure 4:**
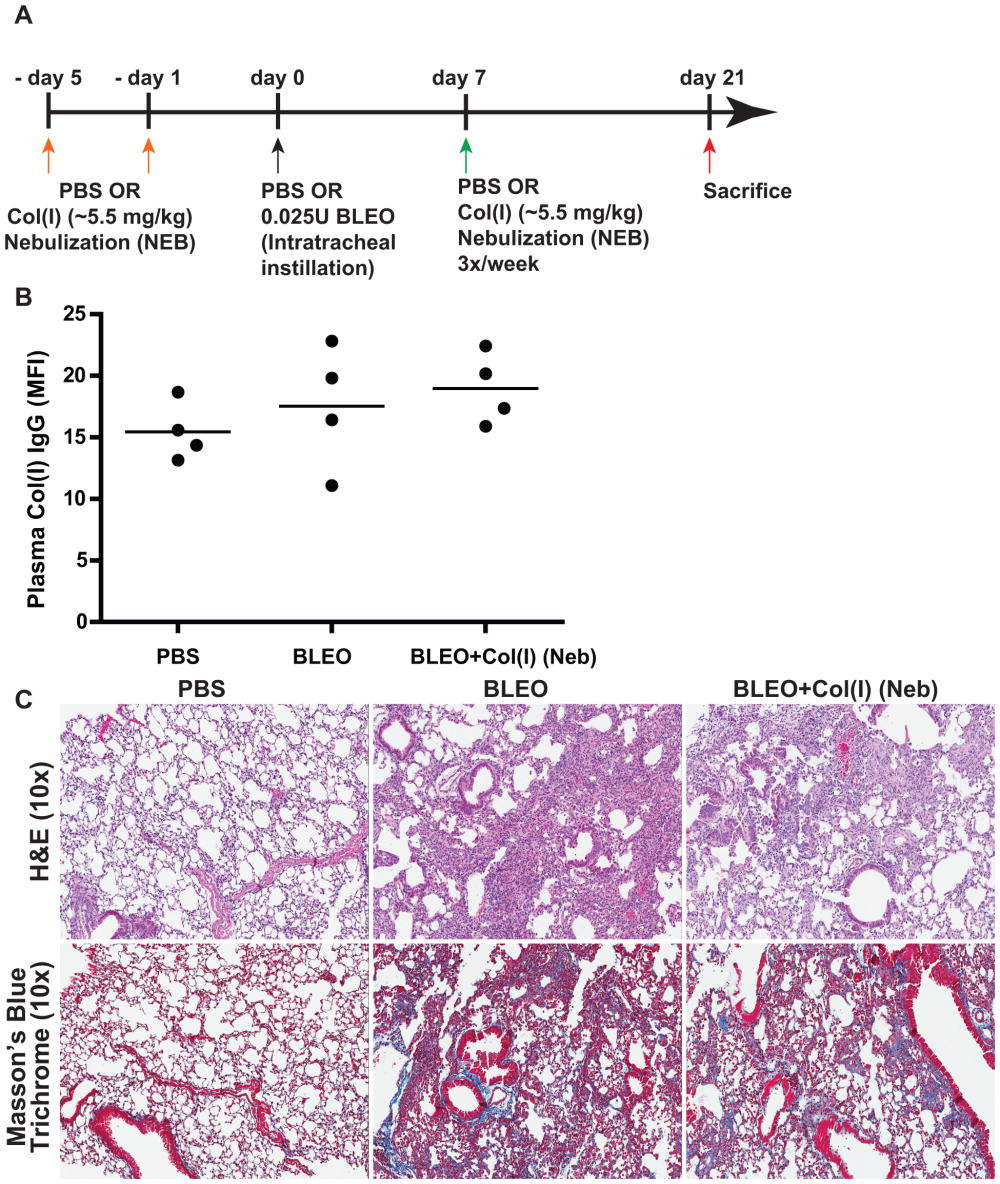
Tolerance induction of Col(I) does not confer protection against bleomycin-induced fibrosis. (**A**) Schematic illustration of the experimental design. (**B**) Circulating antibodies specific to col(I) were unchanged in 21 day post bleomycin injury mice. Values represent mean ± SEM; number of animals: PBS = 4, BLEO = 4 and BLEO+col(I) Neb = 4. (**C**) At day 21 post bleomycin injury, lung tissue sections were stained for H&E and Masson's blue trichrome staining. Extensive collagen deposition was observed with bleomycin injury and col(I) nebulized lungs. Original magnifications: 10×. (Figure S2B: 1×).

Type I collagen [col(I)], is the major lung collagen, and is highly expressed in IPF lungs [27]. However, high levels of circulating anti-col(I) antibodies were not detected in IPF patients (Figure 2) or in bleomycin-induced fibrosis (Figure 4B). Furthermore, nebulizing col(I) did not prevent fibrosis induced by bleomycin (Figure 4C). Our studies suggest that the fibro-protective effects of nebulizing a native lung protein, col(V) in particular, are a specific response.

### Tolerance induction of col(V) suppresses local T lymphocyte proliferation and pro-inflammatory/pro-fibrotic cytokine expression

**Figure 5:**
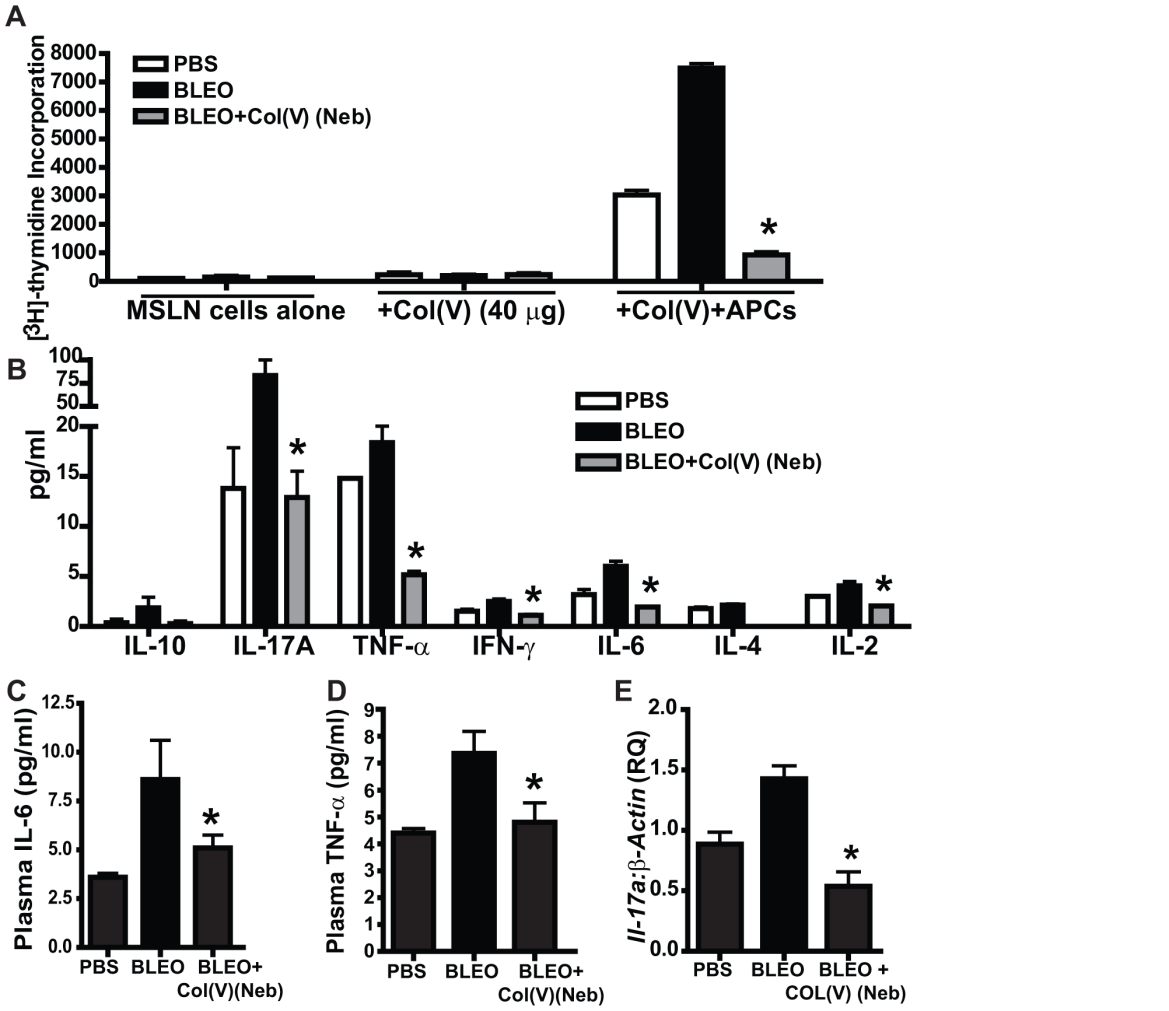
Tolerance induction of col(V) suppresses T lymphocytes activation and associated pro-inflammatory/pro-fibrotic cytokine expression. (**A**) At day 21 post bleomycin injury, mediastinal lymphocytes were isolated and cultured alone or in the presence of autologous antigen presenting cells (APCs from naïve C57-BL/6 mice) for 48 h. The cells were then radiolabeled with tritiated thymidine for 16 h and assessed for proliferation rates. Values represent mean ± SEM. n = 5 mice/group; Compared to bleomycin group, * p<0.01; one-way ANOVA, post hoc test: Bonferroni. (**B**) Conditioned media from A was analyzed for Th1/Th2/Th17 cytokines. Values represent mean ± SEM. n = 5 mice/group; Compared to bleomycin group, * p<0.01; one-way ANOVA, post hoc test: Bonferroni. (**C**, **D**) Plasma samples from (**A**) were analyzed for Th1/Th2/Th17 cytokines. Values represent mean ± SEM. n = 5 mice/group; Compared to bleomycin group, * p<0.01; one-way ANOVA, post hoc test: Bonferroni. (**E**) At day 14 post bleomycin injury, whole lung homogenates from bleomycin-injured mice+col(V) treatment were quantified for *il-17a* mRNA expression and normalized for *β-actin*. Values represent mean ± SEM. n = 5 mice/group; compared to bleomycin group, * p<0.01; one-way ANOVA, post hoc test: Bonferroni.

We have previously demonstrated lung allograft rejection is in part mediated by anti-col(V) immunity and that col(V)-induced tolerance abrogated the rejection response [28] and Wilson and colleagues had reported that bleomycin-induced lung fibrosis may be T cell dependent [29]. We next determined if immune tolerance was the mechanism of col(V)-induced prevention of lung fibrosis we had earlier shown in Figure 3. Data in Figure 5 shows that mediastinal lymph node lymphocytes isolated from col(V)-treated mice do not proliferate in response to col(V) when presented by autologous antigen presenting cells, therefore indicating col(V)-induced tolerance (Figure 5A). IL-17, IL-6, and TNF-α have all been linked to fibrosis [29]–[31]. Therefore, we next examined the cytokines produced in the supernatants of the mixed leukocyte reactions reported in Figure 5A. Notably, col(V)-induced tolerance was associated with IL-17A, IL-6 and TNF-α (Figure 5B). We also noted a reduction in IFN-γ but not IL-10 (Figure 5B). These changes were also observed systemically by data showing lower serum levels of IL-6 and TNF-α (Figure 5C, D), as well as lower lung transcript levels for *Il-17a* in col(V)-treated mice (Figure 5E), albeit at an earlier time point at day 14 wherein we could detect higher mRNA expression of *Il-17a* in bleomycin-injured mice. Collectively, the above data indicates that immune tolerance induced by col(V) protects against bleomycin injury by blunting T cell activation and the associated systemic and local expression of pro-inflammatory and pro-fibrotic cytokines.

### Col(V) prevents collagen accumulation in established fibrosis

**Figure 6:**
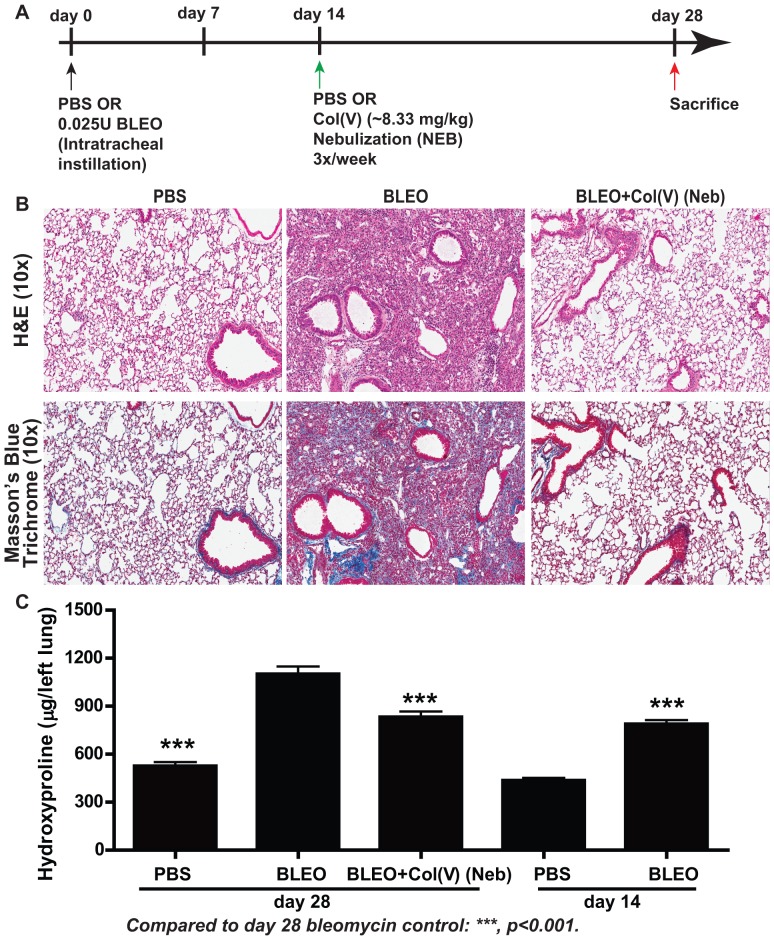
Col(V) treatment protects against established fibrosis. (**A**) Schematic illustration of the experimental design. (**B**) At day 28 post bleomycin injury, lung tissue sections were stained for H&E and Masson's blue trichrome staining. Extensive collagen deposition was observed with bleomycin injury while col(V) nebulized lungs, similar to normal lungs, had collagen deposition around airways and vasculature. Original magnifications: 10×. (Figure S2C: 1×). (**C**) Collagen deposition was measured quantitatively by assaying for hydroxyproline concentrations from the whole left lung day 28 and day 14 post bleomycin injury+col(V) treatment. Values represent mean ± SEM; n = 12 mice/group; * p<0.001 for day 28 bleomycin vs. day 28 PBS and day 28 col(V) (Neb); day 14 bleomycin vs. day 14 PBS; day 28 bleomycin vs. day 14 bleomycin; one-way ANOVA, post hoc test: Bonferroni.

We next determined if nebulized col(V) would arrest ongoing collagen deposition in a model of chronic fibrosis induced by bleomycin. Based on efficacy established in preliminary studies, we nebulized 8.33 mg/kg bodyweight of col(V) protein three times a week beginning at day 14 post bleomycin injury, a time when fibrosis is established [32], followed by sacrifice at day 28 as schematically presented in Figure 6A. Notably, we observed significant protection from fibrosis (Figure 6B; top panel) and attenuated collagen deposition as observed by Masson's trichrome staining for collagen (Figure 6B; lower panel) and hydroxyproline content (Figure 6C). Notably, at the time of nebulization of col(V), there was significantly higher hydroxyproline concentrations in the lung at day 14 post bleomycin instillation compared to PBS-instilled lungs (p<0.001). We then observed that compared to day 14 post bleomycin instillation, at day 28 post bleomycin, the lungs had higher hydroxyproline concentration (p<0.01). Compared to day 28 post bleomycin, col(V) nebulization significantly attenuated hydroxyproline concentrations in the lung (p<0.001). Through our studies, we conclude that delayed administration of col(V) in a nebulized form is effective in arresting the progression of established fibrosis.

### Nebulized col(V) downregulates local fibrosis-related transcripts

**Figure 7:**
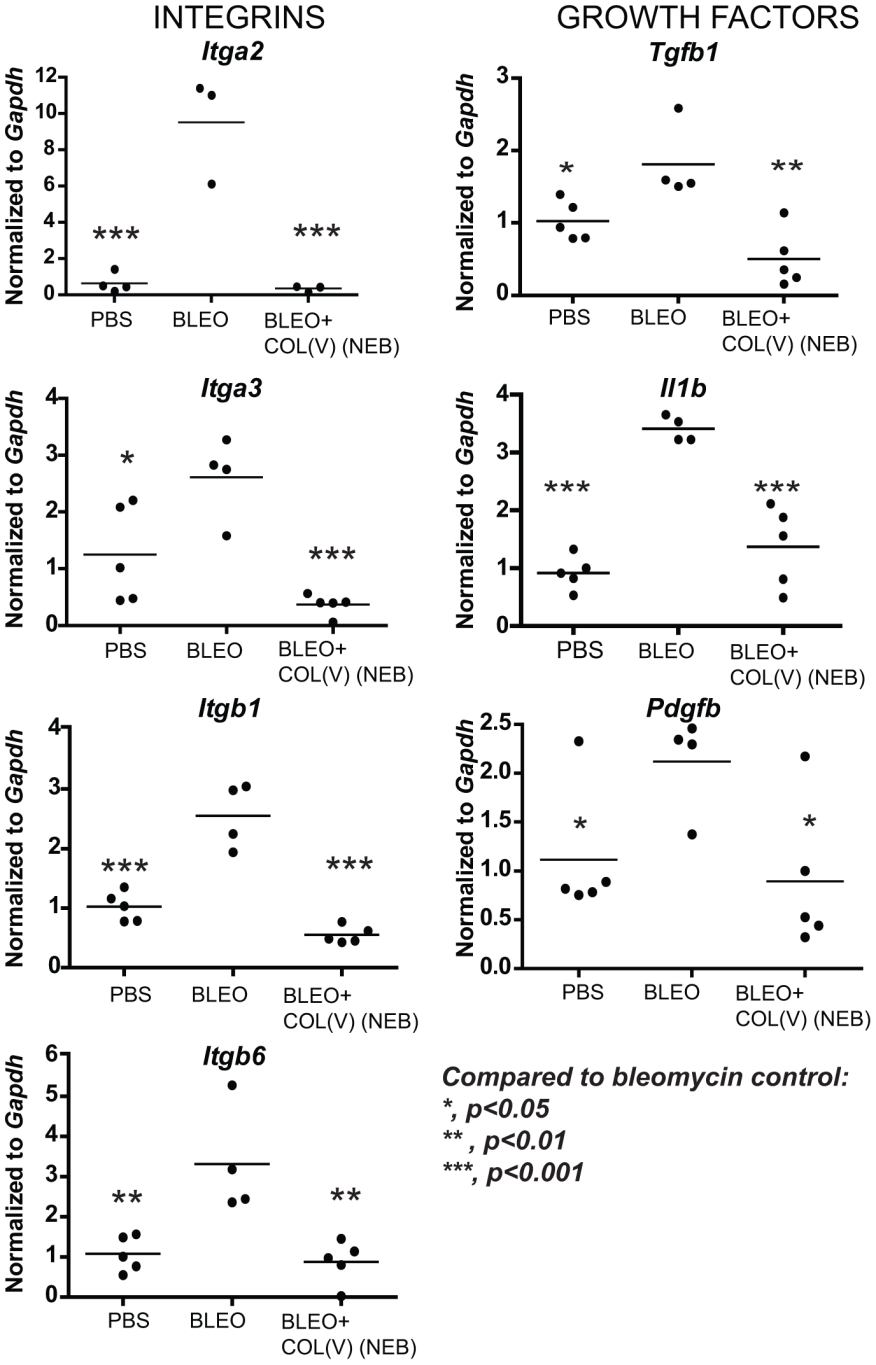
Col(V) treatment downregulates integrins, TGF-β and other pro-fibrotic cytokines. At day 28 post bleomycin injury, lung tissues were homogenized and cDNA was analyzed for fibrosis-specific gene expression analyses.

**Figure 8:**
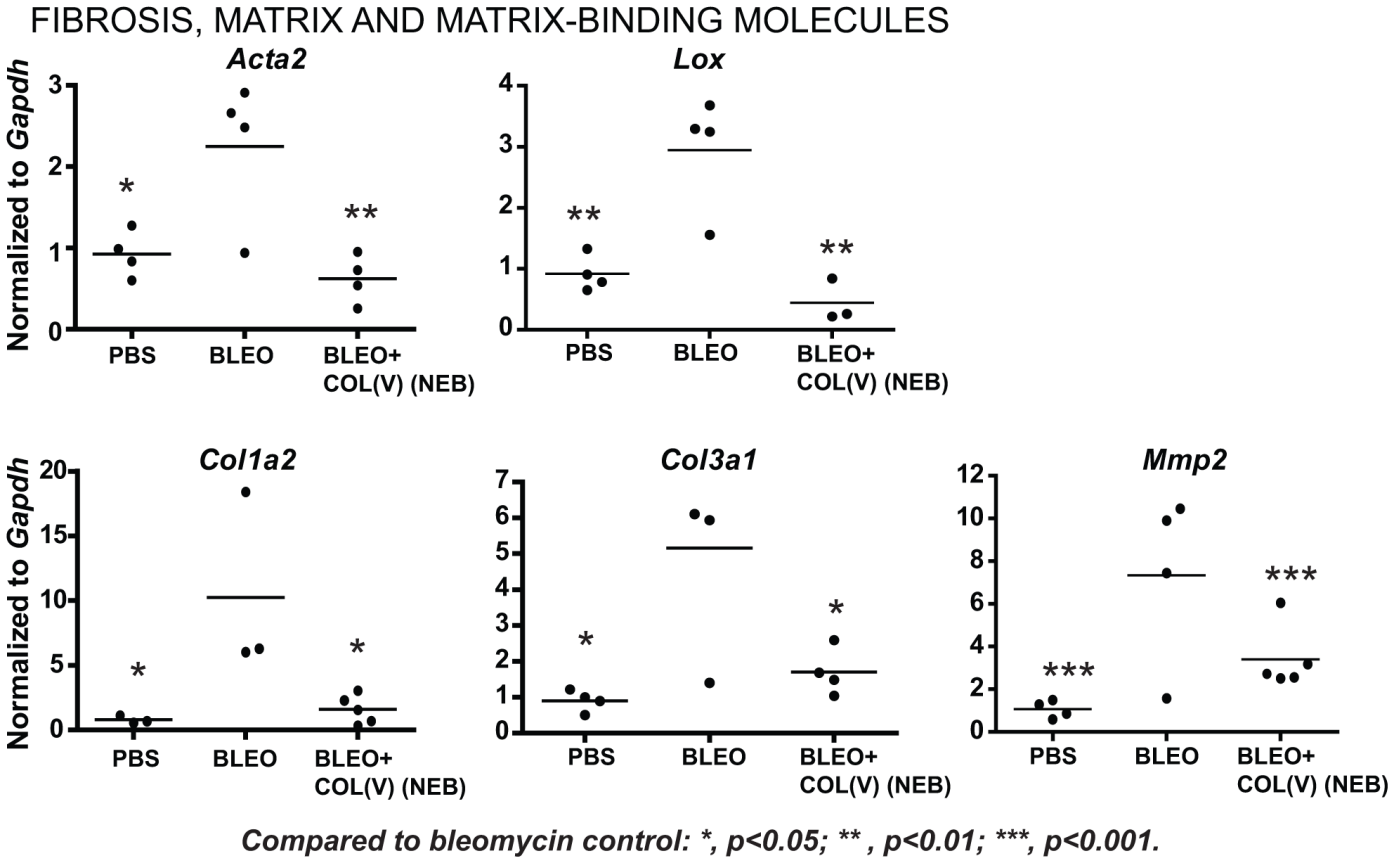
Col(V) treatment downregulates matrix molecules. At day 28 post bleomycin injury, lung tissues were homogenized and cDNA was analyzed for fibrosis-specific gene expression analyses.

**Figure 9:**
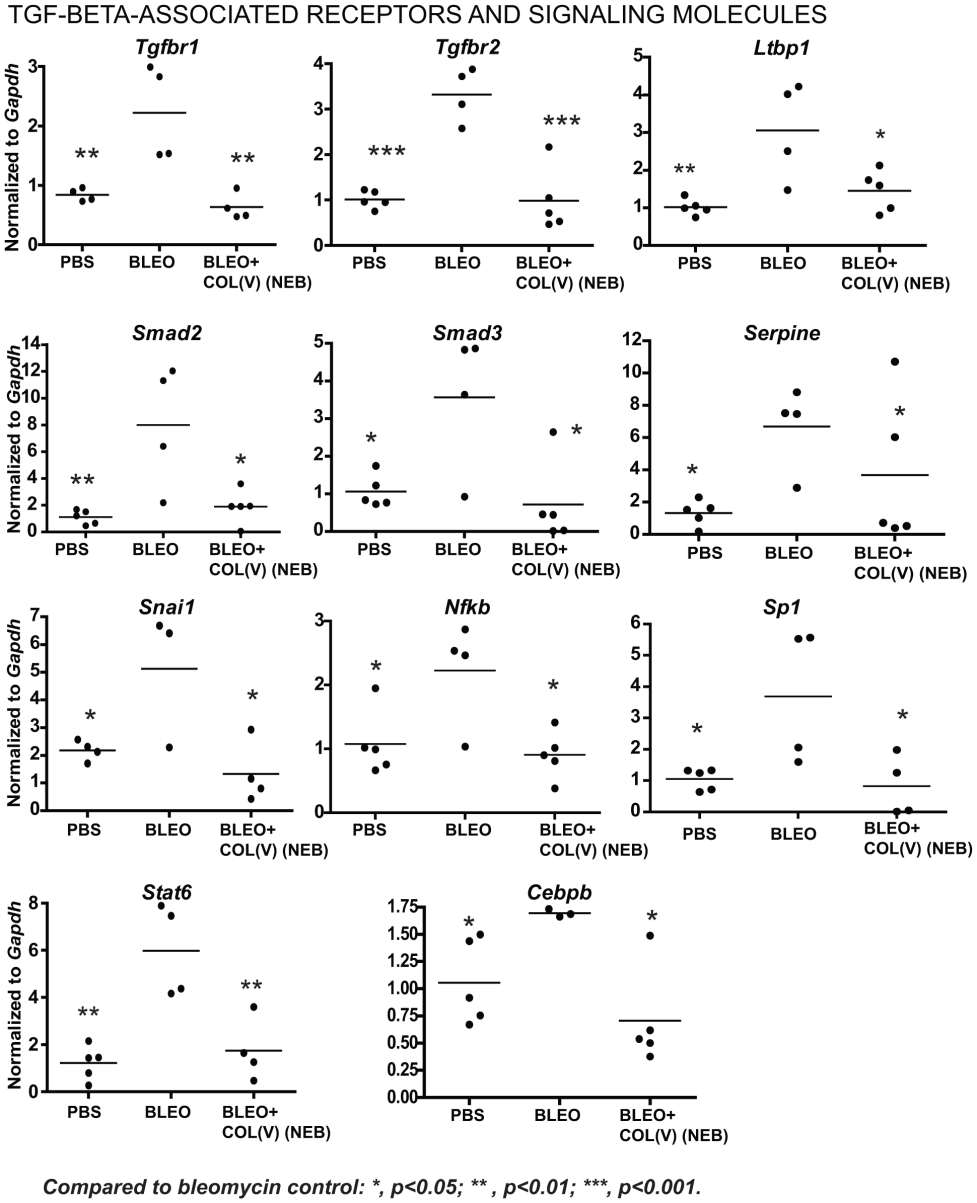
Col(V) treatment downregulates TGF-β associated receptors, signaling molecules and transcription factors. At day 28 post bleomycin injury, lung tissues were homogenized and cDNA was analyzed for fibrosis-specific gene expression analyses.

To further investigate mechanisms underlying the beneficial effects of col(V) in established fibrosis, we next analyzed transcript expression for fibrosis-related genes in the bleomycin model. In a comparison of PBS or bleomycin-instilled lung, we observed upregulation of genes from five major functional classes implicated as fibrogenic factors in IPF (Table 1). Nebulized delivery of col(V), suppressed bleomycin-induced genes listed in the following functional categories: integrins (*Itga1, Itga2, Itgb1, Itgb6*, Figure 7), pro-fibrotic growth factors (*Tgfb, Il1b, Pdgfb*, Figure 7), fibrosis and matrix-related molecules (*Acta2, Col1a2, Col3a1,Mmp2, Lox*, Figure 8) and members of the TGF-beta superfamily (*Tgfbr1/2, Smad2/3, Ltbp1, Serpine1, Nfkb/Sp1/Cebpb*, Figure 9) in addition to *Snai-1* and *Stat6* (Figure 9). These data suggest that treatment with col(V) may lead to attenuation of multiple pro-fibrotic genes involved in the pathogenesis of IPF.

**Table 1:** List of bleomycin-induced genes modulated by col(V) treatment.

		Gene		Bonferroni-adjusted P value	
Functional Class	Unigene	Symbol	Gene Description	Upregulation	Downregulation
				PBS vs BLEO	BLEO vs BLEO+COL(V)
**Integrins**					
	Mm.5007	Itga2	Integrin alpha 2	0.001	0.001
	Mm.57035	Itga3	Integrin alpha 3	0.05	0.001
	Mm.263396	Itb1	Integrin beta 1 (fibronectin receptor beta)	0.001	0.001
	Mm.98193	Itb6	Integrin beta 6	0.01	0.01
**Cytokines/Growth Factors**					
	Mm.248380	Tgfb1	Transforming growth factor, beta 1	0.05	0.01
	Mm.222830	Il1b	Interleukin 1 beta	0.001	0.001
	Mm.144089	Pdgfb	Platelet derived growth factor, B polypeptide	0.05	0.05
**Fibrosis, matrix and matrix-binding molecules**					
	Mm.213025	Acta2	Actin, alpha 2, smooth muscle, aorta	0.05	0.01
	Mm.277792	Col1a2	Collagen, type I, alpha 2	0.05	0.05
	Mm.249555	Col3a1	Collagen, type III, alpha 1	0.05	0.05
	Mm.172	Lox	Lysyl oxidase	0.01	0.01
	Mm.29564	Mmp2	Matrix metallopeptidase 2	0.05*	0.05*
	Mm.439656	Cebpb	CCAAT/enhancer binding protein (C/EBP), beta, latency associated peptide (LAP)	0.05*	0.05*
**Members of the TGF-beta superfamily**					
	Mm.197552	Tgfbr1	Transforming growth factor, beta receptor I, ALK5	0.05	0.01
	Mm.172346	Tgfbr2	Transforming growth factor, beta receptor II	0.001	0.001
	Mm.269747	Ltbp1	Latent transforming growth factor beta binding protein 1	0.01	0.01
	Mm.391091	Smad2	MAD homolog 2 (Drosophila)	0.01	0.05
	Mm.7320	Smad3	MAD homolog 3 (Drosophila)	0.05	0.05
	Mm.250422	Serpine1	Serine (or cysteine) peptidase inhibitor, clade E, member 1, PAI-1	0.05	0.05
**Transcription factors**					
	Mm.2093	Snai1	Snail homolog 1 (Drosophila)	0.05*	0.01*
	Mm.256765	Nfkb	Nuclear factor of kappa light polypeptide gene enhancer in B-cells 1, p105	0.05	0.05
	Mm.4618	Sp1	Trans-acting transcription factor 1	0.05	0.05
	Mm.121721	Stat6	Signal transducer and activator of transcription 6	0.01	0.01

* indicates Newman-Keul's-adjusted P value.

## Discussion

Col(V), a minor collagen in the lung, is sequestered within the fibrils of type I collagen, the major pulmonary collagen. Our studies demonstrate the systemic production of anti-col(V) autoantibodies as a specific response and associated local expression of individual alpha chains of col(V) in clinical IPF specimens. Accordingly, tolerizing mice with nebulized col(V), but not col(I), prevented lung fibrosis and lower production of inflammatory cytokines. The current study also shows that nebulized col(V) protein arrests further development of bleomycin-induced fibrosis by the suppression of multiple fibrosis-related genes, specifically members of the TGF-β superfamily. To the best of our knowledge, the current study is the first to show the efficacy of nebulized therapy of a native protein in the treatment of established fibrosis. mRNA transcript data indicate that the beneficial effect of col(V) likely involves multiple pathways involved in fibrogenesis.

Previous reports by Parra and colleagues describe the role of col(V) in the pathogenesis of IPF by illustrating the distributions of col(V) in two different lung compartments and by using three-dimensional reconstruction to evaluate the amount of collagen [26]. The potential caveat therein may possibly be the green auto fluorescence emitted by lungs resulting in interference of measurements. Our studies demonstrate the overexpression of the individual chains of col(V) proteins in IPF lungs, which was determined by pepsin digestion of the lungs followed by careful systematic extraction of total collagen [22]. The mRNA expression of col(V) was analyzed in IPF tissue sections with confirmed fibrotic lesions. Similarly, our pre-clinical studies provide evidence that nebulized col(V), but not col(I), blunts circulating autoantibodies and renders col(V)-specific immunosuppression with attenuation of associated cytokines. Braun and colleagues examined effects of systemically tolerized col(V) in a bleomycin model of acute lung injury by using measures of inflammation [20]. We present effects of nebulized col(V) in a bleomycin model of chronic fibrosis using hallmarks of bleomycin-induced fibrosis/IPF: a) T cell activation, b) collagen deposition and c) myofibroblast differentiation.

**Figure 10:**
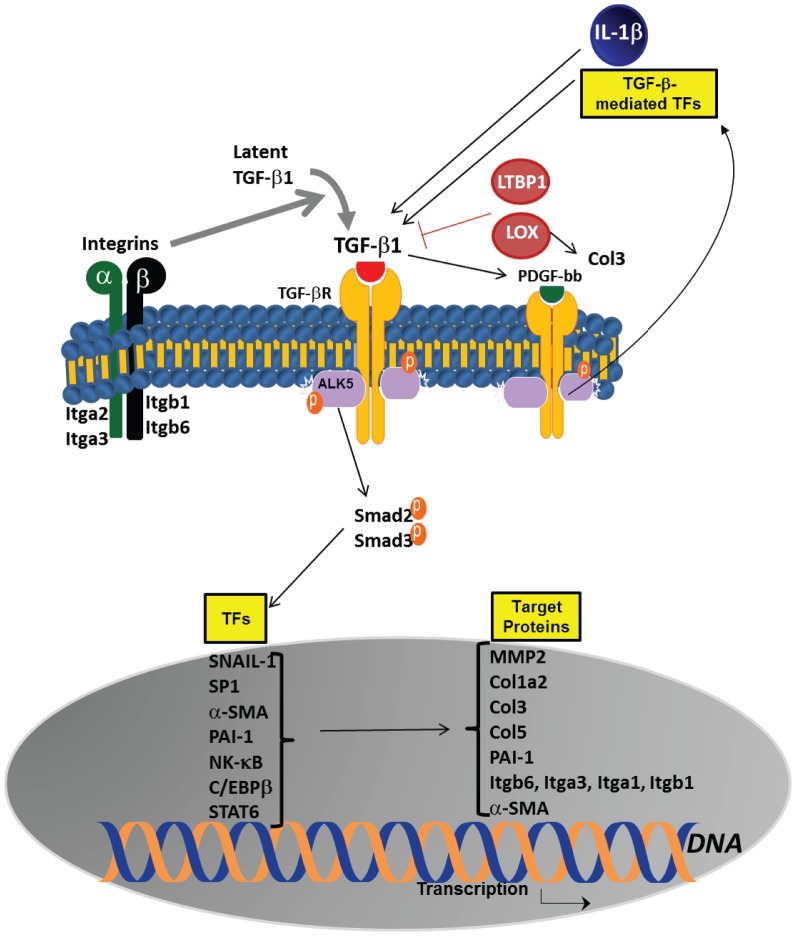
Schematic representation of pathways affected by intrapulmonary instillation of col(V).

In order to simulate the relevant clinical setting wherein lungs of IPF patients at the time of diagnosis are significantly scarred, we used the bleomycin model of established fibrosis. Henderson and colleagues have previously demonstrated the effectiveness of using a therapeutic model examining bleomycin-induced lung fibrosis in which an intervention was administered at the time of established fibrosis to determine if fibrosis could be reversed [32]. Our demonstrate that nebulized col(V) arrests the progression of established fibrosis possibly via mitigation of multiple key fibrotic pathways (Figure 10). However, these pathways appear to be centrally revolving around TGF-β and its associated downstream signaling molecules. Even more significantly, a few integrins, the major receptors for cell adhesion to extracellular matrix proteins and other cells, are downregulated in response to col(V) treatment and are discussed here. αVβ6 integrin (*Itgb6*) activates latent TGF-β and hence lack of this gene [33] or its inhibition [34] protects against lung fibrosis. α3β1 integrin (*Itgb1*) has been implicated in activation of latent TGF-β and hence epithelial-mesenchymal transition [35] and additionally, it facilitates β-catenin-Smad3 crosstalk to promote myofibroblast formation and survival [9]. α2β1 integrin (*Itga2*), a receptor for type I collagen, is reportedly upregulated in response to TNF-α in IPF lung-derived fibroblasts [36].

While bleomycin-induced TGF-β and members of the TGF-β superfamily are suppressed by col(V) nebulization, other pro-fibrotic molecules which crosstalk with TGF-β are suppressed by col(V) and they are discussed below: (i) IL-1β causes acute lung injury via integrin-dependent mechanisms, including αVβ6 integrin [7] and (ii) PDGF signaling pathways [37]. Increased levels of lysyl oxidase (LOX), an amine oxidase critical for the initiation of collagen and elastin cross-linking [38], increased mRNA levels of collagen type I (*Col1a2*), collagen type III (*Col3a1*) and matrix metalloproteinase (*Mmp2*) have been reported in murine models of lung fibrosis [39]. Interestingly, *Serpine1* - also known as the TGF-β-regulated protein PAI-1 – has been reported to directly correlate with collagen accumulation and that the TGF-β/PAI-1 signaling axis plays a vital role in myofibroblast survival [40]. The nuclear transcription factors which are blocked by col(V) treatment and implicated in pulmonary fibrosis are NF-κB [41], Sp1 [42] and CCAAT/enhancer-binding protein β (*Cebpb*) [43], SNAIL [44] and STAT6 [45]. While Velosa et al [18] have demonstrated that col(V)-induced tolerance downregulates TGF-β in an experimental model of systemic sclerosis, they had not investigated other related pathways which might likely fuel the pro-fibrotic signaling. Our studies suggest that col(V) likely confers its fibroprotective effects by targeting integrins, in addition to TGF-β and its associated-signaling pathways. Notably, we detected some bleomycin-induced genes which were unchanged by col(V) treatment and other genes which were unchanged due to bleomycin induction at the determined time of analyses (Figure S3).

In this report, we present evidence which identifies col(V) in an emerging class of target molecules combating specific autoimmune/fibrotic responses. Recent investigations have focused on certain pathologic inflammatory responses which may be due to autoimmunity wherein self-reactive circulating autoantibodies have been detected among patients and associated with acute disease process [46], [47]. While early studies show presence of circulating immune complexes [48]; a recent report has identified mutations in autoimmune regulator gene as a link between autoimmune responses and defective central immunotolerance in IPF [49]. Hence, circulating antibodies to self-antigens have been widely documented but their roles as potential immunotherapy has not been demonstrated. A prior study from our laboratory has identified the localization of col(V) in the apical surface of airway epithelial cells [21]. Interestingly, most of the documented evidence on auto-antibodies has been related to proteins specific to the epithelium which is the primary line of defense against injury due to pro-inflammatory/pro-fibrotic cytokines, pathogenic microbes and other agents such as the reactive oxygen species.

The limitations in this study would call for longitudinal studies in clinical tissues to understand the trend of antibody response relative to disease progression. Similarly, in our mouse models, elucidation of the functional and phenotypic effects of col(V) antibodies and their products on key cell types involved in the pathogenesis of IPF, such as epithelial, endothelial or mesenchymal cells. The interaction site between col(V) and integrins coupled with their functional and phenotypic effects in epithelial and mesenchymal cells are unknown. Therefore, the role of potential signaling molecules unraveled in this study, in addition to the effect of col(V) antibodies on impaired epithelialization, tissue repair and matrix remodeling, which are typical features of IPF, will be the target of our future studies in understanding this antigen.

In summary, we present evidence that autoimmunity to col(V) has a role in the pathogenesis of IPF. These studies also provide further insight for potential mechanisms, particularly TGF-β-related signaling molecules, which may be driving the auto-antigen specific immunosuppression for IPF.

## Supporting Information

Figure S1 Pepsin digested lung homogenates (15 µg) and corresponding standards run in a 5% gel and Coomassie stained. Image shown here is representative of 3 normal and 5 IPF tissues.
(TIF)

Figure S2 **A**. Tolerance induction of col(V) protects against bleomycin-induced fibrosis. H&E and trichrome images of data presented in Figure 3. **B**. Tolerance induction of col(I) does not protect against bleomycin-induced fibrosis. H&E and trichrome images of data presented in Figure 4. **C**. Col(V) treatment protects against bleomycin-induced fibrosis. H&E and trichrome images of data presented in Figure 4. Original magnifications: 1×.
(TIF)

Figure S3 Hierarchical clustergram of all 80 genes modulated by col(V) treatment.
(TIF)

Table S1 Excel file with raw data from the PCR-based gene array is provided in Table S1 (Supplemental Information-5).
(XLS)

File S1 Supplementary Methods.
(DOCX)
