# Correction: Type V Collagen Induced Tolerance Suppresses Collagen Deposition, TGF-β and Associated Transcripts in Pulmonary Fibrosis

**DOI:** 10.1371/journal.pone.0209107

**Published:** 2018-12-06

**Authors:** Ragini Vittal, Elizabeth A. Mickler, Amanda J. Fisher, Chen Zhang, Katia Rothhaar, Hongmei Gu, Krista M. Brown, Amir Emtiazjoo, Jeremy M. Lott, Sarah B. Frye, Gerald N. Smith, George E. Sandusky, Oscar W. Cummings, David S. Wilkes

The eighth author’s name is spelled incorrectly. The correct name is: Amir Emtiazjoo.
