# Supplementary figures and images for: Type V Collagen Induced Tolerance Suppresses Collagen Deposition, TGF-β and Associated Transcripts in Pulmonary Fibrosis
Source: PLoS One. 2013 Oct 21;8(10):e76451. doi: 10.1371/journal.pone.0076451 (PMC3804565; doi:10.1371/journal.pone.0076451)

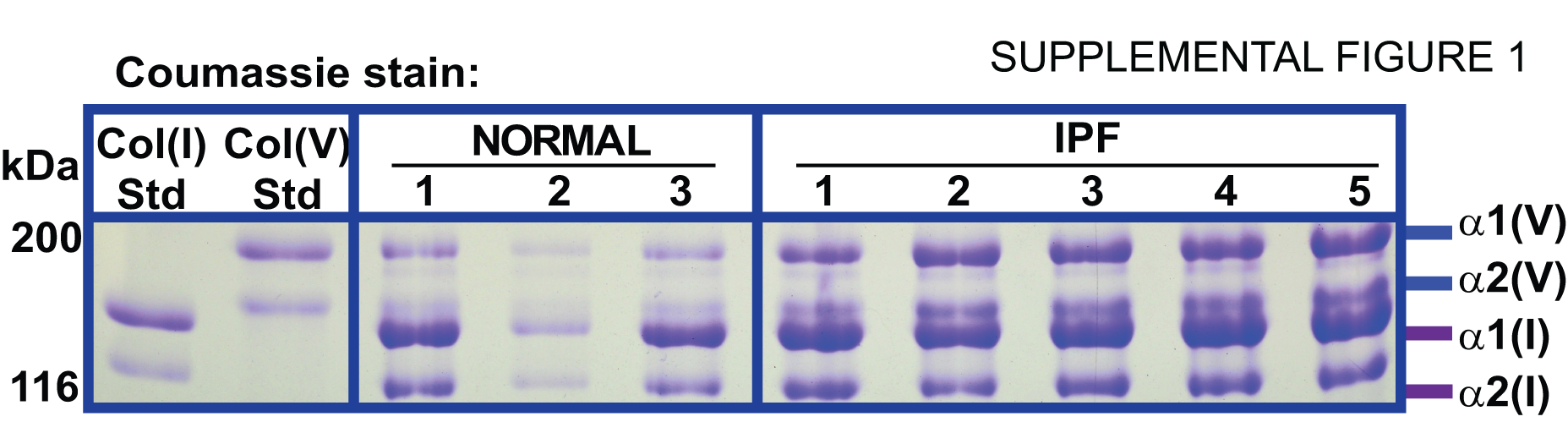

Supplement: Figure S1 — Pepsin digested lung homogenates (15 µg) and corresponding standards run in a 5% gel and Coomassie stained. Image shown here is representative of 3 normal and 5 IPF tissues. (TIF) [file pone.0076451.s001.tif]

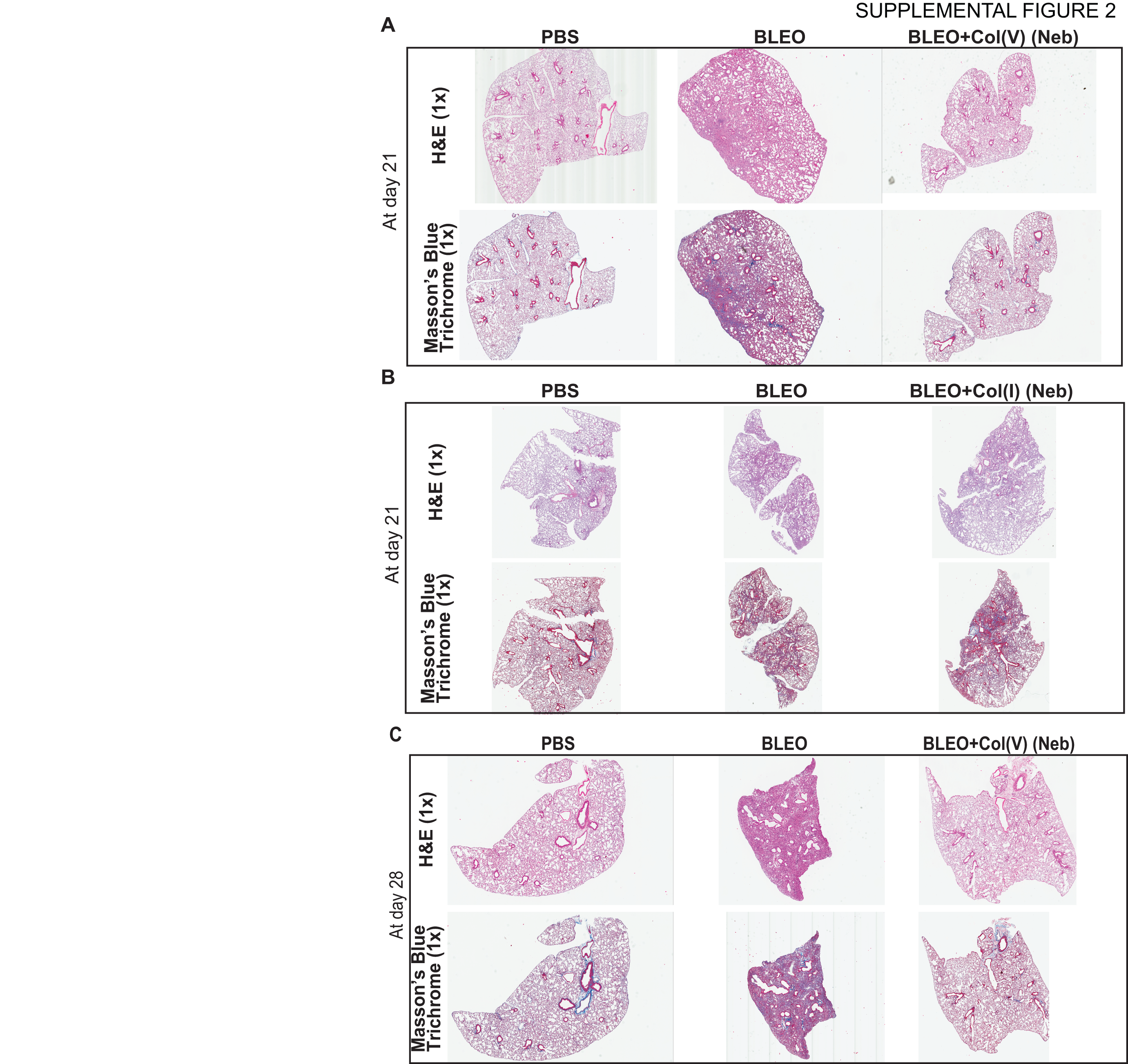

Supplement: Figure S2 — A. Tolerance induction of col(V) protects against bleomycin-induced fibrosis. H&E and trichrome images of data presented in Figure 3. B. Tolerance induction of col(I) does not protect against bleomycin-induced fibrosis. H&E and trichrome images of data presented in Figure 4. C. Col(V) treatment protects against bleomycin-induced fibrosis. H&E and trichrome images of data presented in Figure 4. Original magnifications: 1×. (TIF) [file pone.0076451.s002.tif]

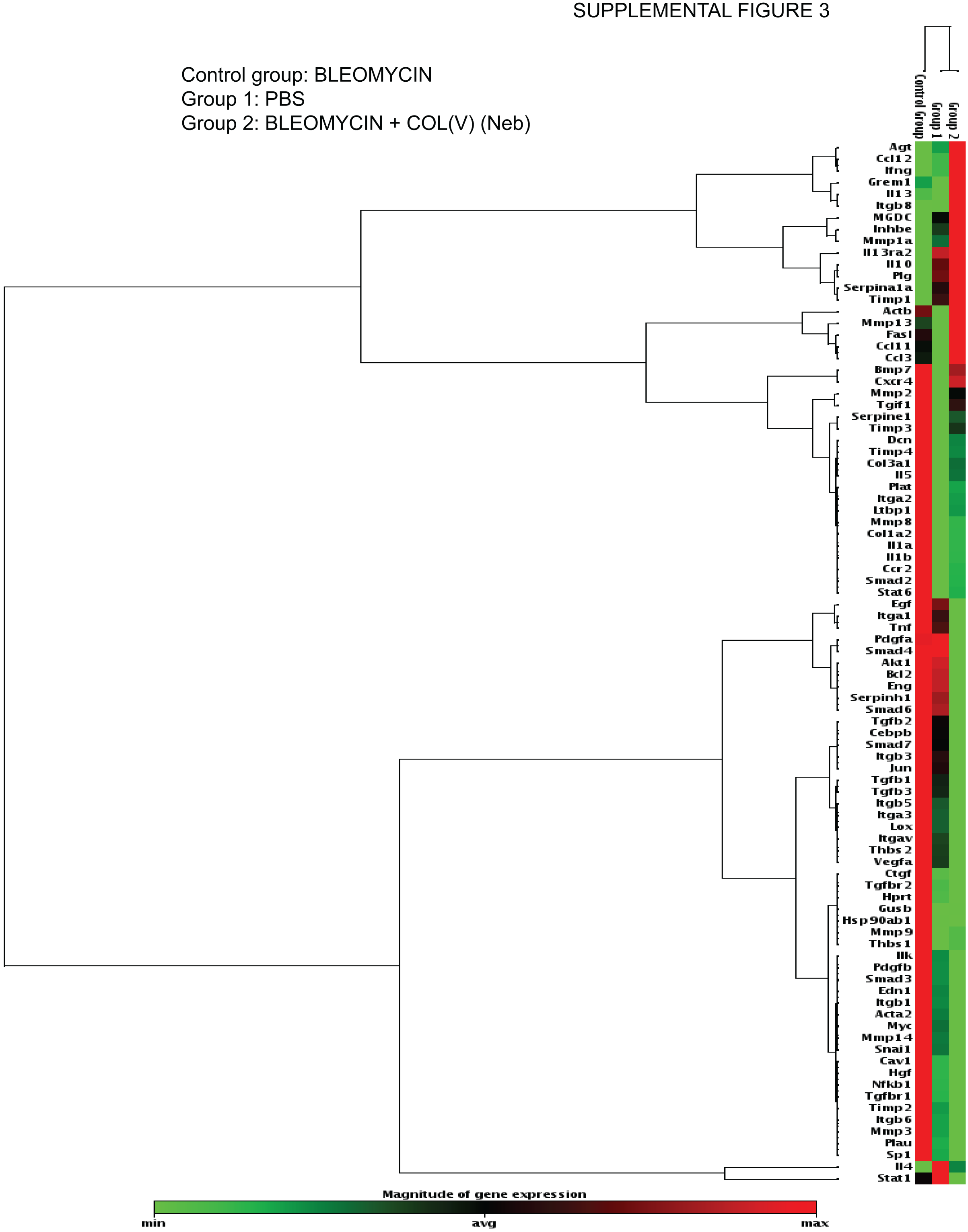

Supplement: Figure S3 — Hierarchical clustergram of all 80 genes modulated by col(V) treatment. (TIF) [file pone.0076451.s003.tif]
