# Supplementary material for: Type V Collagen Induced Tolerance Suppresses Collagen Deposition, TGF-β and Associated Transcripts in Pulmonary Fibrosis
Source: PLoS One. 2013 Oct 21;8(10):e76451. doi: 10.1371/journal.pone.0076451 (PMC3804565; doi:10.1371/journal.pone.0076451)
Supplement: File S1 — Supplementary Methods. (DOCX) [file pone.0076451.s005.docx]

**Methods.**

**Antibodies and other reagents.** The antibodies used for immunoblotting or immunofluorescent labeling are as follows: α-smooth muscle actin (Dako Corp., Carpenteria, CA), col(V) (Lifespan Biosciences, Seattle, WA) and col(I) (Novus Biologicals).

**Immunohistochemistry.** Clinical or murine tissue sections confirmed by board-certified pathologist, were obtained from paraffin-embedded, formalin-fixed lungs underwent antigen retrieval treatment, followed by protein block (1x Power Block; Biogenex, San Ramon, CA). Sections were incubated with primary antibodies specific to col(V) and col(I) (1:50) or α-SMA (1:100) for 1h, and then incubated with rhodamine-conjugated secondary antibodies or with streptavidin-labeled secondary antibodies followed by DAB. The sections were counterstained with DAPI or hematoxylin and imaged using Zeiss microscope.

**Real Time PCR.** Total RNA was isolated from two 20µm scrolls of formalin-fixed paraffin embedded tissue using RecoverAll™ Total Nucleic Acid Isolation Kit (Ambion) according to manufacturer’s instructions. cDNA was synthesized from total RNA using the iScript cDNA synthesis kit (Bio-Rad, Hercules, CA) primed by oligo-dT and random primers; 0.2 µg of total RNA was used for each reaction with a total reaction volume of 20 µl. The reaction mixtures were incubated at 25⁰C for 5 min, 42⁰C for 30 min, and 85⁰C for 5 min. 2 µl of each cDNA product was used for quantitative PCR analysis. Real-time RT-PCR for alpha 1 and 2 chains for col(I) and col(V); and GAPDH were performed using the Assays-on-Demand^TM^ gene expression kits containing two unlabeled PCR primers and a FAM^TM^-labeled Taqman probe (Applied Biosystems, Foster City, CA) on a Smartcycler (Cepheid, Sunnyvale, CA). Target gene expressions were normalized to GAPDH gene expression.

**Hydroxyproline Assay.** Frozen left lung was homogenized and digested overnight in 6N HCl. The supernates were analyzed for hydroxyproline concentrations as previously described ([1](#_ENREF_1)).

**Mixed Lymphocyte Reaction-H^3-^thymidine incorporation.** Lymphocytes were isolated and subjected to proliferation assay as previously described ([2](#_ENREF_2)).

**Cytokine profiling by Cytometric Bead Analysis.** Conditioned media from the mixed lymphocyte reaction was harvested and stored at -80⁰C until analysis. Plasma from the col(V) tolerized mice were collected and stored at -80⁰C until analysis. Cytokine expression levels of IL-17A, IL-10, TNF-α, IFN-γ, IL-6, IL-4 and IL-2 were measured using the mouse Th1/Th2/Th17 Cytokine kit (BD Biosciences, San Jose, CA) as per manufacturer’s instructions.

**Supplemental Figure Legends.**

**Figure S1.** Pepsin digested lung homogenates (15 µg) and corresponding standards run in a 5% gel and Coomassie stained. Image shown here is representative of 3 normal and 5 IPF tissues.

**Figure S2. A.** Tolerance induction of col(V) protects against bleomycin-induced fibrosis. H&E and trichrome images of data presented in Figure 3. **B.** Tolerance induction of col(I) does not protect against bleomycin-induced fibrosis. H&E and trichrome images of data presented in Figure 4. **C.** Col(V) treatment protects against bleomycin-induced fibrosis. H&E and trichrome images of data presented in Figure 4. Original magnifications: 1x.

**Figure S3.** Hierarchical clustergram of all 80 genes modulated by col(V) treatment.

**Table S1.** Excel file with raw data from the PCR-based gene array is provided in Table S1 (Supplemental Information-5).

**References**

1. Hecker L, Vittal R, Jones T, Jagirdar R, Luckhardt TR, Horowitz JC, Pennathur S, Martinez FJ, Thannickal VJ. Nadph oxidase-4 mediates myofibroblast activation and fibrogenic responses to lung injury. *Nature medicine* 2009;15:1077-1081.

2. Yoshida S, Haque A, Mizobuchi T, Iwata T, Chiyo M, Webb TJ, Baldridge LA, Heidler KM, Cummings OW, Fujisawa T, Blum JS, Brand DD, Wilkes DS. Anti-type v collagen lymphocytes that express il-17 and il-23 induce rejection pathology in fresh and well-healed lung transplants. *Am J Transplant* 2006;6:724-735.
